# Targeting STARD4/EGFR axis inhibits growth and overcomes lenvatinib resistance in hepatocellular carcinoma

**DOI:** 10.1016/j.gendis.2025.101556

**Published:** 2025-02-15

**Authors:** Mengting Liu, Yixin Liu, Jiahui Zheng, Xiangping An, Jiayong Wen, Fengchi Zhu, Jin Jia, Dan Guo, Nana Chen

**Affiliations:** a Department of Pharmacy, Nanfang Hospital, Guangdong Provincial Key Laboratory of New Drug Screening, School of Pharmaceutical Sciences, Southern Medical University, Guangzhou, Guangdong 510515, China; b Clinical Pharmacy Center, Nanfang Hospital, Guangdong Provincial Key Laboratory of New Drug Screening, School of Pharmaceutical Sciences, Southern Medical University, Guangzhou, Guangdong 510515, China; c Laboratory of Anti-inflammatory and Immunomodulatory Pharmacology, Guangdong Provincial Key Laboratory of New Drug Screening, School of Pharmaceutical Sciences, Southern Medical University, Guangzhou, Guangdong 510515, China

**Keywords:** EGFR/PI3K/AKT pathway, Hepatocellular carcinoma, Invasion, Lenvatinib resistance, Migration, Proliferation, STARD4

## Abstract

Lenvatinib is widely used as a first-line chemotherapy for advanced hepatocellular carcinoma (HCC), a highly metastatic and recurrent cancer. However, HCC cells often develop resistance to lenvatinib, thus reducing its efficacy. This study aims to investigate the impact of STARD4, a crucial cholesterol transporter, on HCC growth and lenvatinib resistance, as well as explore the involvement of the EGFR/PI3K/AKT signaling pathway in STARD4's role. Analysis of clinical samples from HCC patients revealed increased expression of both STARD4 and EGFR in tumor tissues, with a strong correlation between STARD4 expression and malignancy progression. *In vitro* and *in vivo* studies demonstrated that STARD4 promoted HCC growth and hindered lenvatinib's anti-tumor effect, while STARD4 down-regulation exerted opposite effects. Further investigation revealed that depletion of STARD4 increased cholesterol accumulation in the plasma membrane, resulting in reduced EGFR phosphorylation. Moreover, cholesterol depletion attenuated these effects, suggesting STARD4 activates EGFR/PI3K/AKT signaling in a cholesterol-dependent manner. To elucidate the underlying mechanism of lenvatinib resistance, we established the lenvatinib-resistant HCC cell lines and found increased stimulation of both STARD4 and EGFR signaling. Furthermore, the EGFR inhibitor erlotinib suppressed the promotion of HCC progression by STARD4, reinforcing its role in activating the EGFR/PI3K/AKT pathway. In conclusion, this study demonstrates that STARD4 enhances HCC growth and lenvatinib resistance by regulating cholesterol homeostasis and activating the EGFR/PI3K/AKT pathway. These findings suggest STARD4 as a potential molecular biomarker for predicting lenvatinib resistance and as a therapeutic target in HCC treatment.

## Introduction

Hepatocellular carcinoma (HCC) is recognized as the fifth most widespread malignancy across the globe in terms of occurrence and mortality rate.^1^ Clinically, most HCC patients are diagnosed at advanced stages and have poor prognosis due to high metastasis and recurrence probabilities. As a result, molecular targeted therapy has become an essential treatment strategy for advanced HCC patients.^2^ Lenvatinib, a small-molecule inhibitor of multiple receptor tyrosine kinases, is among the first-line therapies but has only limited clinical benefit owing to drug resistance.^2^^,^^3^ Hence, a thorough understanding of the underlying mechanisms that trigger HCC metastasis and drug resistance is critical in identifying novel and promising strategies for HCC treatment.

Metabolic reprogramming is a hallmark of cancer.^4^ The reprogramming of cholesterol metabolism is essential for energy provision, membrane organization, and protumorigenic signaling throughout cancer progression.^5^ Cholesterol transport and biotransformation, as well as dysregulation of *de novo* cholesterol biosynthesis, have been identified in multiple cancer cells.^6^ Previous studies have indicated that a significant portion of sterol transport occurs through highly efficient non-vesicular mechanisms.^7^^,^^8^ The steroidogenic acute regulatory protein (StAR)-related lipid-transfer (START) domain family is known to play a crucial role in the non-vesicular transport of cholesterol between membranes.^9^ Among these proteins, STARD4, which is transcriptionally regulated by the sterol-responsive element-binding protein 2 (SREBP-2), stands out as an important player in maintaining cholesterol homeostasis.^10^ Previous studies have identified STARD4 as highly expressed in various cell types, including mouse fibroblast line 3T3-L1, human THP-1 macrophages, Kupffer cells, and hepatocytes. Its function involves transferring cholesterol to the endoplasmic reticulum, which is rich in acyl-CoA cholesterol acyltransferase-1 (ACAT-1).^11^ Through this process, STARD4 facilitates the production of cholesteryl ester, contributing to the maintenance of intracellular cholesterol homeostasis.^11^^,^^12^ STARD4 silencing leads to a reduced rate of sterol transport to the endocytic circulation chamber and cholesteryl esterification.^13^ Previous studies have additionally observed that knockdown of STARD4 attenuates the cholesterol-mediated regulation of SREBP-2 activation and perturbs lipid homeostasis.^14^ To date, limited research has been conducted regarding the functions of STARD4 in tumors. STARD4 has been found to be abnormally up-regulated in certain tumors, including head and neck squamous cell carcinoma^15^ and prostate cancer,^16^ and has been implicated in the malignant progression of breast cancer.^17^^,^^18^ A recent study has revealed that the SREBF2-STARD4 axis confers resistance to sorafenib in HCC by regulating mitochondrial cholesterol homeostasis.^19^ However, the specific actions of STARD4 in HCC progression and the underlying mechanisms remain unclear and warrant further investigation.

Lenvatinib is an oral multitarget tyrosine kinase inhibitor that primarily targets vascular endothelial growth factor receptors, platelet-derived growth factor receptor α (PDGFRα), fibroblast growth factor receptors, RET, and KIT.^20^, ^21^, ^22^, ^23^ Its efficacy as a first-line treatment for patients with unresectable HCC has been demonstrated in a multicenter, randomized phase 3 trial.^24^ Specifically, the trial revealed that lenvatinib achieved non-inferiority in overall survival and superiority in progression-free survival, compared with sorafenib, in the context of untreated advanced HCC. Even so, the overall response rate for lenvatinib is only 24.1%, underscoring the imperative to investigate the underlying mechanisms of drug resistance and enhance therapeutic efficacy.^24^ Emerging studies have shown that activation of the epidermal growth factor receptor (EGFR) plays a significant role in lenvatinib resistance. In HCC cells with high levels of EGFR expression, treatment with lenvatinib results in feedback activation of the EGFR-P21 activated kinase 2 (PAK2)-extracellular signal-regulated kinase 5 (ERK5) signaling axis,^25^ while another study illustrates that HCC cells acquire lenvatinib resistance through activating the EGFR-signal transducer and activator of transcription 3 (STAT3)-ATP binding cassette subfamily B member 1 (ABCB1) pathway.^26^ However, the mechanism of EGFR activation triggering lenvatinib resistance remains largely unknown and deserves further investigation.

There is increasing evidence supporting the role of cholesterol in the formation of lipid rafts which regulates the oncogenic signaling pathway.^27^ Notably, depletion of cholesterol from lipid rafts triggers both EGFR ligand-dependent and independent dimerization and phosphorylation, which suggests that cholesterol in lipid rafts and caveolae inhibits EGFR activation and signaling.^28^^,^^29^ However, some studies suggest a supporting role of cholesterol in EGFR activation.^30^ Specifically, cholesterol has been shown to reduce internalization of EGFR and promote activation of extracellular signal-regulated kinase 1/2 (ERK1/2) pathway in prostate cancer.^31^ To date, it remains unclear whether and how cholesterol influences the activation of the EGFR signaling in HCC.

As STARD4 plays crucial roles in maintaining intracellular cholesterol homeostasis, the objective of the current study is to investigate the effects of STARD4 on the growth and resistance of HCC to lenvatinib and to elucidate whether EGFR activation is involved in the action of STARD4.

## Materials and methods

### Cell culture

Human hepatic cell line L02, human HCC cell lines (Huh7, SMMC 7721, Bel-7402, HCC LM3, SK-Hep-1, HepG2), and human embryonic kidney cells (HEK-293T) were obtained from the Cell Bank of the Chinese Academy of Sciences (Shanghai, China), while human HCC cells (PLC/PRF/5) cells and human umbilical vein endothelial cells (HUVEC) were purchased from the American Type Culture Collection (ATCC). Human embryonic kidney cell line HEK-293T was also included in the study. All cell lines were cultured in high-glucose Dulbecco's modified Eagle medium (DMEM, Gibco, USA) supplemented with 10% fetal bovine serum at 37 °C in a 5% CO_2_ incubator.

To establish lenvatinib-resistant (LR) cell lines, HepG2 or Huh7 cells were treated with increasing concentrations of lenvatinib (MedChemExpress, USA), starting from 1 μM. The cell culture media were refreshed every 48 h until the cells reached 90% confluence, at which point they were passaged. After replating, the lenvatinib concentration was incrementally increased by 1 μM until the cells exhibited rapid proliferation at a higher concentration. This process took at least 6–7 months to establish and maintain the cells in a medium containing a certain concentration of lenvatinib (20 μM for HepG2 and 10 μM for Huh7). The resulting LR cell strains were designated as HepG2-LR or Huh7-LR.

### Clinical specimens

All samples of human HCC tissues and adjacent tissues were collected from patients who underwent surgical procedures at Nanfang Hospital of Southern Medical University between June and September 2023. The freshly obtained tissues were either stored in liquid nitrogen or fixed with 4% paraformaldehyde. Before sample collection, all patients provided written informed consent, and the study protocol received approval from the Medical Ethics Committee of Nanfang Hospital, Southern Medical University.

### Cell transfection

According to the product manual, the cells were transfected with small interfering RNA (siRNA) against STARD4 (target sequence: UGGUCAGCUUUGGAAUAUAA) or overexpression plasmids of STARD4 (pCDH-CMV-MCS-EF1-copGFP-T2A-Puro) using Lipofectamine™ RNAimax (Invitrogen, USA) or Lipofectamine™ 2000 (Invitrogen, USA), respectively. After 6 h of transfection, the medium with transfection reagent was removed and cells were cultured for 24–48 h until further assay.

For the establishment of stable cell lines, lentiviral plasmids encoding STARD4 or shRNA (PLKO-U6-GFP-Puro), as well as packaging plasmids psPAX2 and pMD2.G, were co-transfected into HEK-293T cells using Lipofectamine™ 2000. 48 h after transfection, the lentivirus-containing supernatant was collected for subsequent infection of PLC/PRF/5 cells. Next, the PLC/PRF/5 cells were then subjected to puromycin (Biosharp, China) selection at a concentration of 4 μg/mL until stable survival was achieved. The synthesis and construction of plasmids were performed by Kidan Biosciences (Guangzhou, China).

### Cell viability assay

Cell viability was examined utilizing the CCK8 method. Cells were seeded in 96-well plates and allowed to attach overnight. After different treatments, cell viability was assessed using the CCK8 reagent (Fdbio science, China), according to the manufacturer's protocol. Optical density values were measured at 450 nm using a microplate spectrometer (Tecan, Austria). Cell viability was determined using the following formula: Cell proliferation rate (%) = experimental optical density value/control optical density value × 100. IC_50_ values were calculated by GraphPad Prism.

### Colony formation assay

Approximately 8 × 10^2^ cells were seeded into each well of a 12-well plate. After 10 days of culture, the cells were fixed with 4 % paraformaldehyde and stained with 1 % crystal violet dye (Solarbio, China). Subsequently, the cells were photographed and counted by an ELISPOT analyzer (CTL, USA).

### Wound healing assay

The cells were seeded at a density of 4 × 10^5^ cells per well in 12-well plates and allowed to reach 90%–100% confluency overnight. Subsequently, three straight wounds were created by scratching the cells using a pipette tip. The wounds were then gently washed with phosphate buffer saline solution and the cells were cultured in a medium containing 2% fetal bovine serum. Microscopic images of cell migration were captured at 0 h and 48 h after the scratches were made. The migratory activity was quantified by measuring the migration area within the scratches in each well using Image J software. The migration rate was calculated as the ratio of the relative migration area in the experimental group to that in the control group.

### Transwell assay

The migration and invasion ability of cells was assessed using the transwell chamber (8 μM, Corning, USA). For the invasion assay, Matrigel (BioCoat, USA) was applied to coat the upper chambers. Cells were suspended in a serum-free medium and placed in the upper compartment, while DMEM containing 15% fetal bovine serum was added to the bottom compartment. 24 h after incubation, the migrated or invaded cells were fixed with 4% paraformaldehyde and stained with 1% crystal violet dye for 15 min. At least three random fields were captured for each insert, and cell counts were conducted using an inverted light microscope (Axio Observer A1; ZESS, Germany) at 10 × magnification. The migration rate and invasion rate were quantified by calculating the ratio of the number of migrated or invaded cells in the experimental group to those in the control group, respectively.

### Tube formation assay

Briefly, HUVEC cells were suspended in the culture supernatant of transfected HCC cells and subsequently seeded onto a 96-well plate coated with Matrigel. Following an incubation period of 6–8 h, images of the tube formation were captured using a microscope (Axio Observer A1; ZESS, Germany), and the number of nodes was quantified using Image J software.

### RNA extraction and quantitative reverse transcription PCR

All RNA samples were extracted according to the manufacturer's protocol. mRNA reverse transcription was completed employing an Evo M-MLV RT Premix (ACCURATE BIOLOGY, China), and quantitative PCR was conducted using Taq Pro universal SYBR qPCR Master Mix (Vazyme, China). Results were analyzed with the LightCycler 480 system (Roche, Germany). β-Actin or 18S served as the internal reference, and the relative expression level of the target gene was determined using the 2^−ΔΔCt^ method. The primer sequences used are shown in Table 1.

**Table 1:** Primer sequence for quantitative reverse transcription PCR analysis.

Gene	Primer	Sequence (5′–3′)
STARD4	Forward	CTCTACAAAGCCCAAGGTG
	Reverse	TCATCAAGCTGTCCCAATC
EGFR	Forward	AGGCACGAGTAACAAGCTCAC
	Reverse	ATGAGGACATAACCAGCCACC
β-actin	Forward	TGGCACCCAGCACAATGAA
	Reverse	CTAAGTCATAGTCCGCCTAGAAGCA
18S	Forward	GTAACCCGTTGAACCCCATT
	Reverse	CCATCCAATCGGTAGTAGCG

### Western blot analysis

All lysates were obtained from cells or tissues using RIPA buffer containing protease and phosphatase inhibitors. Quantified protein lysates were resolved by SDS-PAGE, electro-transferred to PVDF membranes (Merck Millipore, USA), and then probed with primary antibodies against the target proteins. After incubation with secondary antibodies, blots were visualized using the BLT GelView 6000 Pro Imaging System. The antibodies used were present as follows: anti-STARD4 (Abcam, Ab202060; 1:1000 dilution), anti-EGFR (Abclonal, A11575; 1:1000 dilution), anti-phosphorylated EGFR (anti-p-EGFR, Abclonal, AP0992; 1:1000 dilution), anti-phosphoinositide 3-kinase (PI3K) (Abmart, T40115F; 1:1000 dilution), anti-phosphorylated PI3K (anti-p-PI3K, CST, 4228T; 1:1000 dilution), anti-protein kinase B (AKT) (CST, 4691S; 1:1000 dilution), anti-phosphorylated AKT (anti-p-AKT, Abclonal, AP0637; 1:1000 dilution), and β-actin (Fdbio science; 1:1000 dilution).

### Immunofluorescence assay

In general, 1500 cells were seeded into a 96-well plate and fixed with 4% paraformaldehyde for 30 min. Subsequently, following permeabilization with 0.5% Triton X-100 solution, cells were blocked in 10% goat serum for 1 h and then incubated with primary antibodies of anti-STARD4 (Abcam, Ab202060; 1:100 dilution) and anti-p-EGFR (Abmart, T55232S; 1:100 dilution) at 4 °C overnight. Cells were then treated with a secondary antibody (Goat Anti-Rabbit IgG H&L (Alexa Fluor® 594); Abcam, Britain) at room temperature for 2 h. Nuclei were counterstained with DAPI for 5 min followed by imaging using a microscope (ECLIPSE Ti2-E; Nikon, Japan).

### Filipin III staining

In general, 10,000 cells were seeded in a confocal dish and fixed with 4% paraformaldehyde for 10 min. The fixed cells were subsequently stained with filipin III (MedChemExpress, USA) at a final concentration of 50 μg/mL in phosphate buffer saline solution, and the staining was allowed to proceed at room temperature in the dark for 60 min. Following staining, the cells were washed three times and then examined using a confocal microscope (AX R with NSPARC; Nikon, Japan) with an excitation wavelength range of 340–380 nm and an emission wavelength range of 385–470 nm.

### Immunohistochemistry assay

The paraffin-embedded tissues were mounted on glass slides and subsequently subjected to deparaffinization and hydration. Endogenous peroxidase activity was quenched, and a serum-blocking step was performed at room temperature. The slides were then incubated at 4 °C overnight with primary antibodies against STARD4 (Invitrogen, PA561644; 1:50 dilution), Ki67 (Abcam, Ab16667; 1:200 dilution), p-EGFR (Abmart, T55232S; 1:50 dilution), p-PI3K (CST, 4228T; 1:100 dilution), and p-AKT (Abclonal, AP0637; 1:100 dilution). Subsequently, the slides were washed and a biotinylated secondary antibody was then added to the slides for incubation. Finally, the tissues were stained with DAB solution, dehydrated with ethanol, and mounted. Light microscopy was employed to capture images and analyze the samples.

### Animal studies

Male BALB/c nude mice aged 5–6 weeks were procured from GemPharmatech (Nanjing, China) to investigate the *in vivo* impact of STARD4 on HCC growth. The mice were randomly assigned to two groups (*n* = 7 per group). Following this, 2 × 10^6^ stable transfected PLC/PRF/5 cells with STARD4 overexpression (STARD4-OE) or its negative control (Control) were subcutaneously injected into the right flank of the mice. The growth rate of subcutaneous tumors was determined by measuring tumor volume daily using a vernier caliper. To examine the *in vivo* effect of STARD4 on lenvatinib resistance, male BALB/c nude mice were allocated randomly into four groups (*n* = 6 per group). Two individual groups were established for each cell, wherein 2 × 10^6^ stable transfected PLC/PRF/5 cells with STARD4 knockdown (sh-STARD4) or its negative control (sh-NC) were subcutaneously injected into the right flank of the mice. Tumor volume and body weight were assessed daily. Once the tumor reached a volume of approximately 20 mm^3^, the STARD4 knockdown and control mice were administered oral doses of physiological saline or lenvatinib (10 mg/kg) daily. After 21 days, the mice were euthanized, and the subcutaneous tumors were extracted and weighed. The tumors were subsequently fixed in 4% paraformaldehyde for immunohistochemistry assay. Tumor volume was calculated using the following equation: Tumor volume = (length × width^2^)/2. All animal experiments were conducted in accordance with the guidelines and regulations approved by the Ethics Committee of Animal Experiments of Southern Medical University.

### Statistical analysis

Statistical analyses were conducted using GraphPad Prism version 8.0 (GraphPad Software, CA, USA). The measured data were expressed as mean ± standard deviation. Student's *t*-tests or one-way ANOVA were employed for comparing two or multiple groups, respectively. Counting data were compared using the Chi-squared test or Fisher's exact test. Statistical significance was determined when the *P*-value was less than 0.05 (*P* < 0.05).

## Results

### STARD4 is up-regulated in HCC

**Figure 1:**
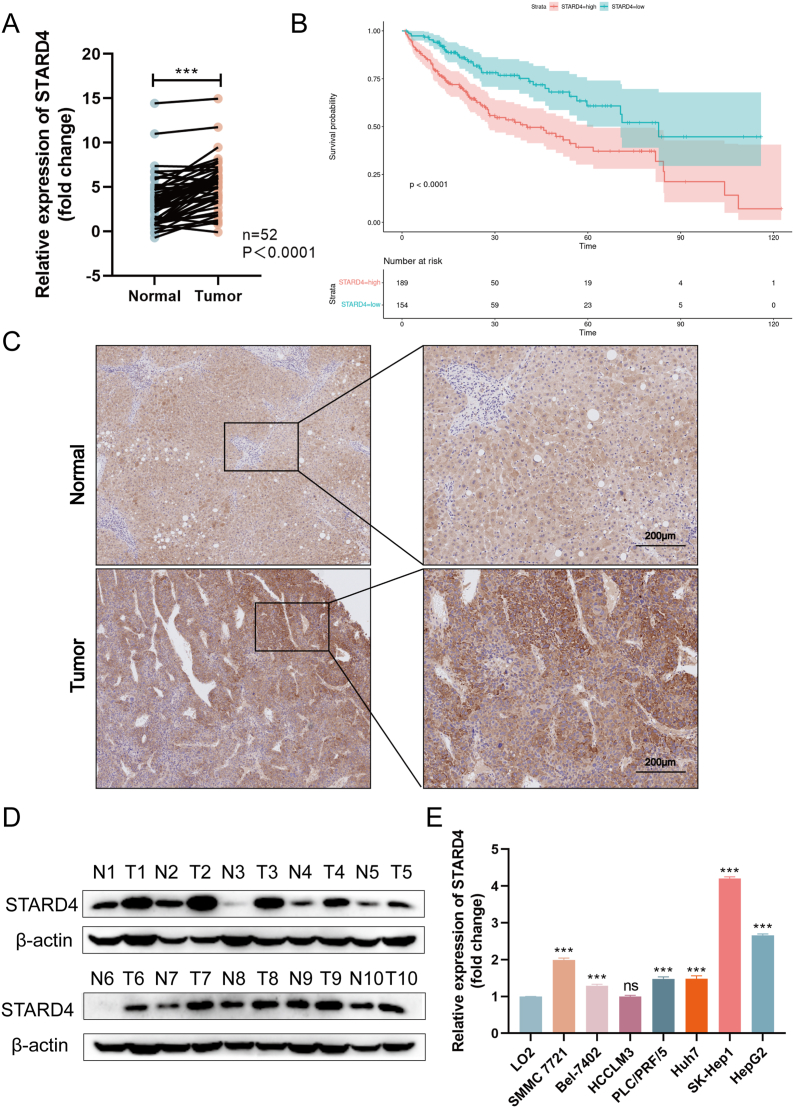
Up-regulation of STARD4 expression in hepatocellular carcinoma tissues and cells. **(A)** Quantitative reverse transcription PCR indicated the expression of STARD4 in 52 pairs of hepatocellular carcinoma tissues and adjacent normal tissues. **(B)** Survival analysis of hepatocellular carcinoma patients with high or low level of STARD4 expression via TCGA data. **(C)** Immunohistochemistry detection of protein abundance of STARD4 in hepatocellular carcinoma tissues and adjacent normal tissues. **(D)** Western blot indicated STARD4 expression in 10 pairs of tumor and para-tumor tissue. **(E)** The relative expression of STARD4 in normal liver cell line and 7 strains of hepatocellular carcinoma cells was detected by quantitative reverse transcription PCR. The data were shown as mean ± standard deviation (*n* = 3). ∗*P* < 0.05, ∗∗*P* < 0.01, and ∗∗∗*P* < 0.001 versus the corresponding control.

To evaluate the role of STARD4 in HCC, we first analyzed its expression in HCC tissues and the matched non-tumor liver tissues. Quantitative reverse transcription PCR revealed that among 52 clinical samples from patients diagnosed with HCC, 46 pairs of tumor tissues showed significant up-regulation of STARD4 mRNA expression, with only 6 pairs showing down-regulation (Fig. 1A). Kaplan–Meier plot from TCGA (The Cancer Genome Atlas) identified an advanced association of the mRNA levels of STARD4 with the overall survival time of the HCC patients (Fig. 1B). Meanwhile, results from immunohistochemistry assays showed that STARD4 was widely distributed in the cytoplasm, and the protein levels in tumor tissue was higher than that in adjacent normal tissues (Fig. 1C). Additionally, western blotting was used to detect the protein levels of STARD4 in 10 pairs of these samples. It was found that STARD4 was significantly up-regulated in tumor tissues compared with the nearby tissues (Fig. 1D). Similarly, in comparison with the human liver cell line LO2, higher mRNA expression of STARD4 was shown in HCC cells including Huh7, SMMC 7721, Bel-7402, SK-HEP-1, HepG2, and PLC/PRF/5 (Fig. 1E). Taken together, the above results indicate that the expression of STARD4 is abnormally up-regulated in HCC tissues and cells, which may be involved in the malignant progression of liver cancer.

### STARD4 is associated with the clinical malignant progression of HCC

To further investigate the correlation between the expression of STARD4 and the clinicopathological characteristics of HCC, we collected surgical resection samples and corresponding clinical data from 52 patients diagnosed with HCC. The samples were divided into a high expression group (26 cases) and a low expression group (26 cases) according to the median of relative mRNA level of STARD4 and the relationship between the expression of STARD4 and other clinical features of HCC patients was analyzed. The analysis results (Table S1) revealed that the mRNA expression level of STARD4 was significantly correlated with tumor size (*P* = 0.032), alpha-fetoprotein levels (*P* = 0.032), hepatitis (*P* = 0.005), TNM stage (*P* < 0.001), and microvascular infiltration (*P* < 0.001). The above analytical results indicate that the increased expression of STARD4 is highly related to the malignant progression of HCC, which suggests that STARD4 could serve as a biomarker for predicting outcomes in HCC patients.

### STARD4 stimulates the proliferation, migration, and invasion of HCC cells

**Figure 2:**
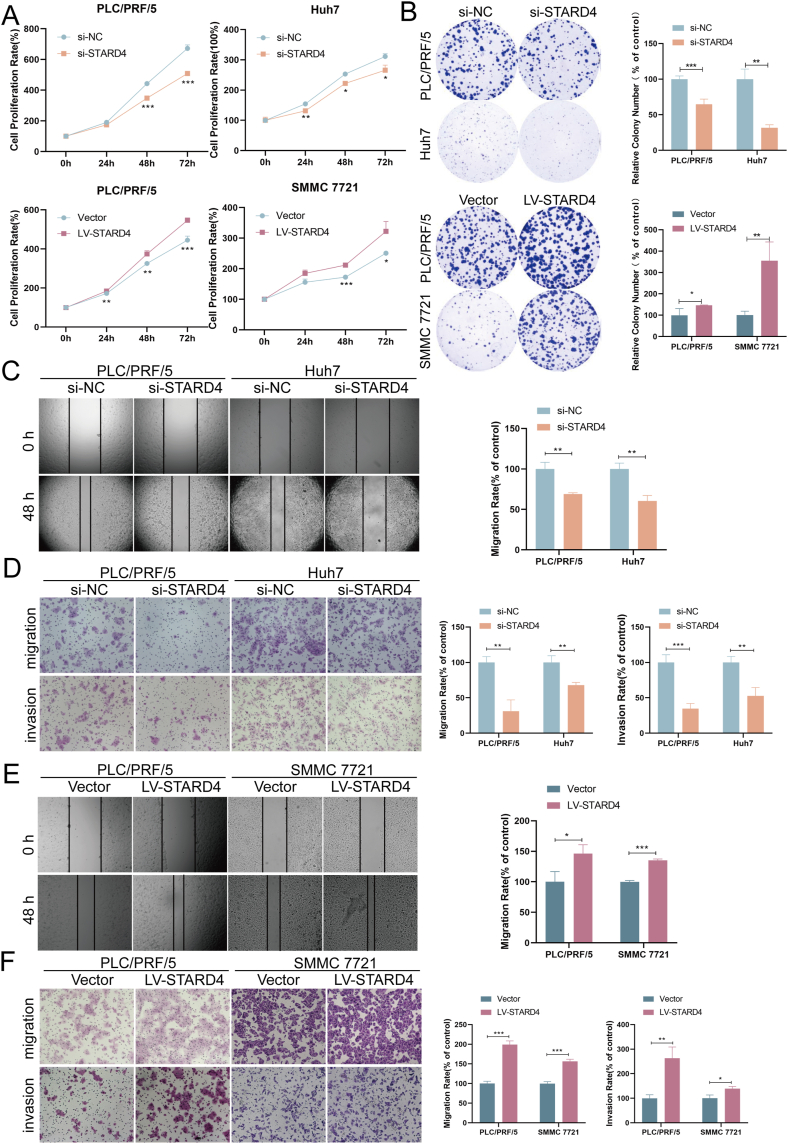
STARD4 stimulates proliferation, migration, and invasion of hepatocellular carcinoma cells. **(A, B)** Cell viability and proliferation of STARD4 silencing (si-NC) or overexpressing (LV-STARD4) hepatocellular carcinoma cells were determined by CCK8 assay and colony formation assay. **(C, D)** Wound healing and transwell assay examined the effects of STARD4 knockdown on migration and invasion of hepatocellular carcinoma cells. **(E, F)** Effects of STARD4 overexpression on migration and invasion of hepatocellular carcinoma cells evaluated by wound healing and transwell assay. The data were shown as mean ± standard deviation (*n* = 3). ∗*P* < 0.05, ∗∗*P* < 0.01, and ∗∗∗*P* < 0.001 versus the corresponding control.

To explore the biological functions of STARD4 in HCC, we investigated the cell viability, colon formation, and migratory and invasive capacity in STARD4 knockdown or overexpressing HCC cells. Results from quantitative reverse transcription PCR and western blotting indicated that the designed siRNA against STARD4 (si-STARD4) or overexpression plasmid (LV-STARD4) could effectively knock down or enhance the mRNA and protein levels of STARD4 in HCC cells (Fig. S1A–D). CCK8 assays revealed that STARD4 knockdown decreased viability of PLC/PRF/5 and Huh7 cells (Fig. 2A). Colony formation assays also confirmed that knockdown of STARD4 inhibited proliferation of PLC/PRF/5 and Huh7 cells (Fig. 2B). Wound healing assay and transwell assay showed that silencing of STARD4 significantly suppressed migratory and invasive capacity of PLC/PRF/5 and Huh7 cells (Fig. 2C, D). Moreover, when STARD4 was overexpressed in PLC/PRF/5 and SMMC 7721 cells, the proliferation (Fig. 2A, B), migration, and invasion (Fig. 2E, F) abilities were obviously enhanced. In addition, decreased tube formation was obtained with HUVECs that were cultivated in the culture supernatant from STARD4 knockdown HCC cells compared with those that grew in the culture supernatant from control cells (Fig. S1E, F). Taken together, these findings indicate that STARD4 functions as a tumor-promoting factor to enhance the proliferation, migration, invasion, and angiogenesis of HCC cells.

### STARD4 activates the EGFR/PI3K/AKT signaling pathway in HCC cells in a cholesterol-dependent manner

**Figure 3:**
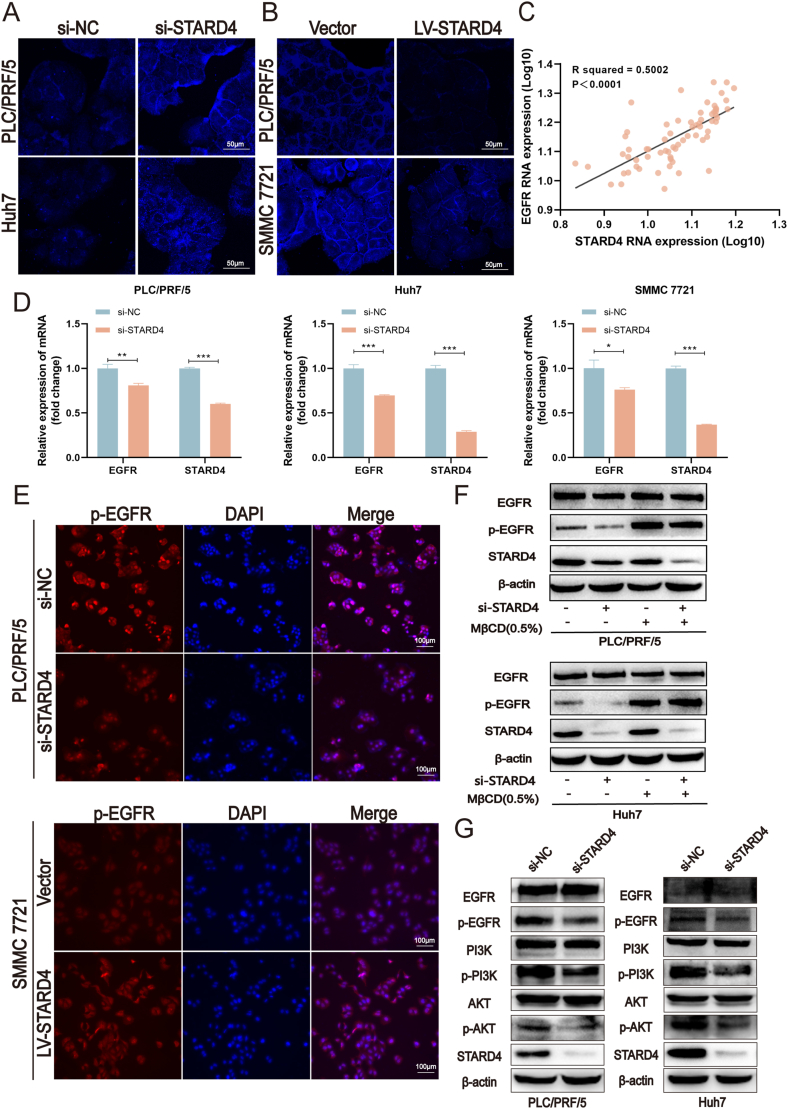
STARD4 activates the EGFR/PI3K/AKT signaling pathway in hepatocellular carcinoma cells in a cholesterol-dependent manner. **(A, B)** Free cholesterol levels of STARD4 overexpressing or silencing hepatocellular carcinoma cells were determined by filipin III staining assays. **(C)** Quantitative reverse transcription PCR showed a correlation between STARD4 and EGFR mRNA expression in clinical patient samples. **(D)** Quantitative reverse transcription PCR detected mRNA expression of STARD4 and EGFR in STARD4 silencing hepatocellular carcinoma cells. **(E)** Immunofluorescence detected the protein level of p-EGFR in STARD4 overexpressing or silencing hepatocellular carcinoma cells. **(F)** The protein levels of STARD4 and p-EGFR in hepatocellular carcinoma cells were added with MβCD after STARD4 silencing. **(G)** The protein levels of STARD4, p-EGFR, p-PI3K, and p-AKT in STARD4 knockdown hepatocellular carcinoma cells were determined by Western blot. The data were shown as mean ± standard deviation (*n* = 3). ∗*P* < 0.05, ∗∗*P* < 0.01, and ∗∗∗*P* < 0.001 versus the corresponding control.

To investigate the effect of STARD4 on the intracellular cholesterol of HCC, we performed filipin III staining assays in STARD4 knockdown and STARD4 overexpressing cells. The results showed that compared with the control cells, the filipin staining intensity in STARD4 knockdown cells increased, reflecting an increase in intracellular free cholesterol levels, especially on the plasma membrane, while STARD4 overexpression showed the opposite result (Fig. 3A, B). The previous studies demonstrated that depletion of cholesterol from the plasma membrane triggered EGFR dimerization and phosphorylation but delayed endocytosis,^29^ which motivated us to determine whether STARD4 could promote EGFR signaling. A significant positive correlation between STARD4 and EGFR mRNA expression was found in tumor tissue samples obtained from HCC patients (Fig. 3C). Reduced mRNA levels of EGFR in STARD4 knockdown cells were also discovered by quantitative PCR (Fig. 3D). Immunofluorescence experiment revealed that EGFR phosphorylation decreased in STARD4 knockdown HCC cells but increased in STARD4 overexpressing cells (Fig. 3E). To evaluate whether cholesterol contributed to the influences of STARD4 on the activation of EGFR, we used methyl-β-cyclodextrin (MβCD) to eliminate cholesterol in the cell membrane, to further explore the effect of STARD4 on EGFR phosphorylation. The results showed that the phosphorylation of EGFR was increased in both the control and STARD4 knockdown cells after the addition of MβCD, but the effect of STARD4 knockdown on the phosphorylation of EGFR was no longer significant after MβCD treatment (Fig. 3F), suggesting that STARD4 could regulate the phosphorylation of EGFR by affecting cholesterol in the plasma membrane. The PI3K/AKT signaling is the key downstream pathway of EGFR which regulates tumor cell growth and apoptosis resistance to chemotherapy, as well as cell invasion and migration. The abnormal activation of the PI3K/AKT pathway resulted in drug resistance to tyrosine kinase inhibitors in HCC patients.^32^ Our study indicated that the silencing of STARD4 could inhibit the phosphorylation of EGFR, PI3K, and AKT (Fig. 3G). In summary, the above results suggest that STARD4 affects the activation of the EGFR/PI3K/AKT signaling pathway by regulating the level of cholesterol in the plasma membrane of HCC cells.

### STARD4 promotes tumorigenesis *in vivo*

**Figure 4:**
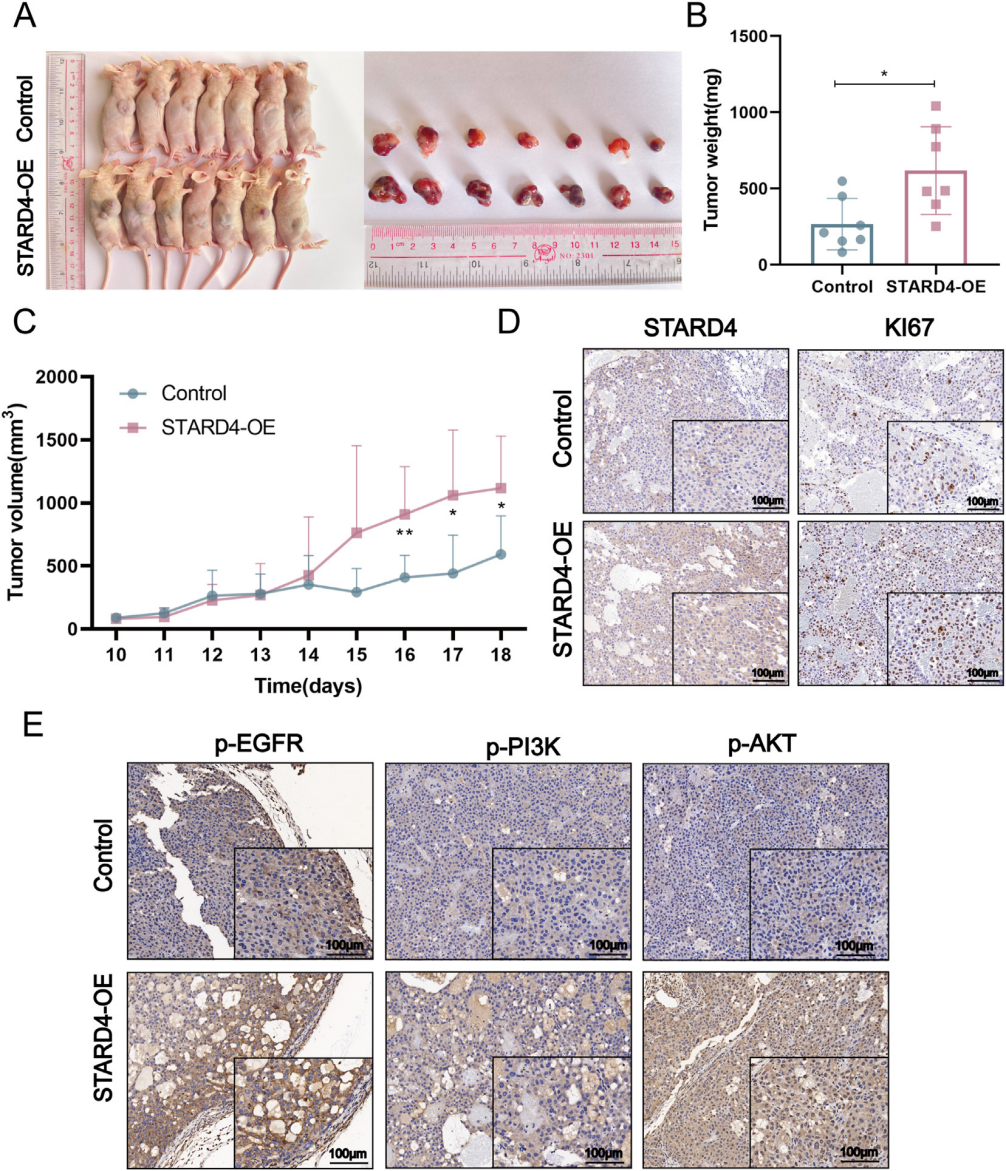
Up-regulation of STADR4 advances the formation of xenograft tumors in nude mice. **(A)** The images showed larger tumors in STARD4 overexpression groups. **(B, C)** Effects of STARD4 overexpression on the tumor volume and tumor weight of the nude mice. **(D)** Immunohistochemistry staining detected the protein level of STARD4 and KI67 in the tumor tissues from the nude mice. **(E)** Immunohistochemistry analysis showed the protein level of p-EGFR, p-PI3K, and p-AKT in the tumor tissues from the nude mice. The data were shown as mean ± standard deviation (*n* = 7). ∗*P* < 0.05, ∗∗*P* < 0.01, and ∗∗∗*P* < 0.001 versus the corresponding control.

To further validate the biological function of STARD4 *in vivo*, we randomly divided 14 nude mice into 2 groups, and STARD4 overexpressing stable cells of PLC/PRF/5 and its negative control cells were injected subcutaneously into the right flank of the nude mice to establish the xenograft model. The obviously elevated STARD4 mRNA and protein levels in the STARD4 overexpressed stable cell line (STARD4-OE) had been confirmed by quantitative reverse transcription PCR and Western blot assays (Fig. S2A, B). Tumor volume and tumor weight were measured, and the data showed that the tumor growth rate of the STARD4 overexpression group (STARD4-OE) was significantly higher than that of the control group (Fig. 4A–C). Immunohistochemistry analysis showed that KI67 expression increased in STARD4 overexpressed tumors compared with the control group (Fig. 4D), indicating that STARD4 exerted a promoting effect on tumor growth. To further investigate the role of STARD4 in the activation of EGFR/PI3K/AKT pathways, we detected the protein level of p-EGFR, p-PI3K, and p-AKT in the tumor tissues from the xenograft nude mice model. Enhanced phosphorylation of EGFR, PI3K, and AKT were displayed in STARD4 overexpression tumor tissues (Fig. 4E). Taken together, the above data demonstrate that STARD4 promotes tumorigenesis *in vivo*.

### Both STARD4 and EGFR signaling are stimulated in acquired lenvatinib-resistant HCC cells

**Figure 5:**
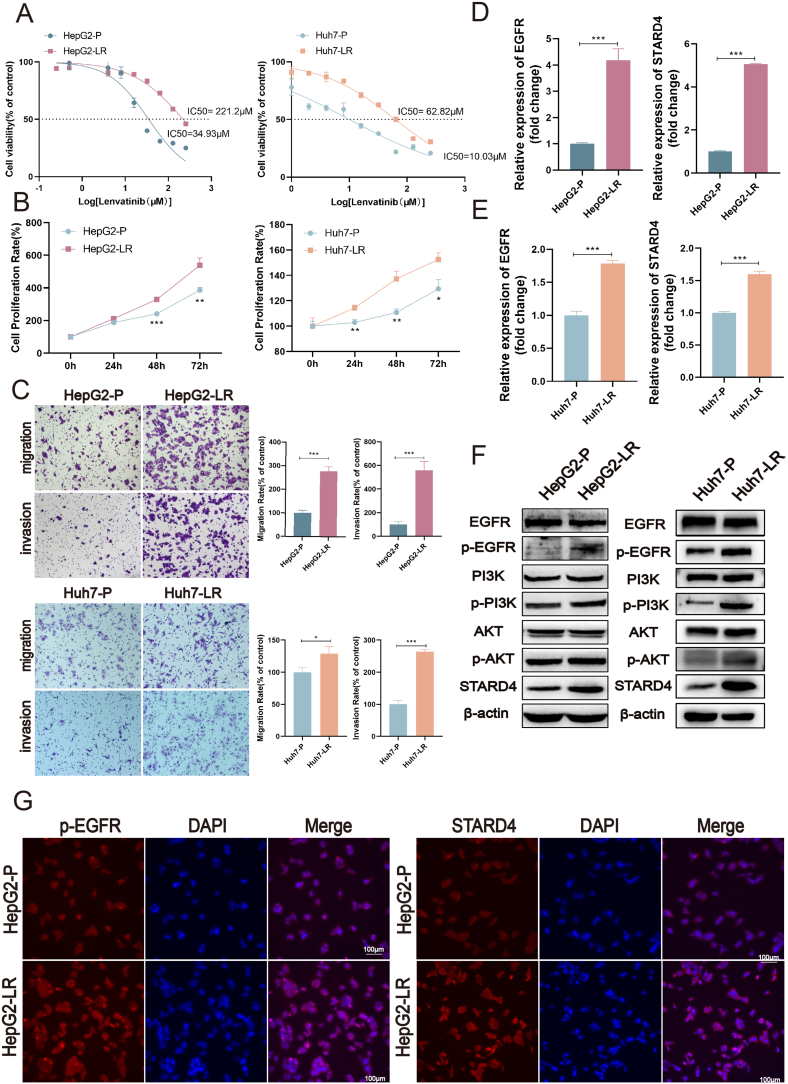
Both STARD4 and EGFR signaling are stimulated in lenvatinib-resistant hepatocellular carcinoma cells. **(A)** The cell viability of HepG2-P, HepG2-LR, Huh7-P, and Huh7-LR cells treated with different concentrations of lenvatinib was assessed by CCK8 assay. **(B)** The cell proliferation rate of HepG2-P, HepG2-LR, Huh7-P, and Huh7-LR cells treated with 50 μM or 10μM of lenvatinib was detected by CCK8 assay. **(C)** Transwell assay examined the migration and invasion of HepG2-P, HepG2-LR, Huh7-P, and Huh7-LR cells treated with 50 μM or 10μM of lenvatinib. **(D)** The mRNA expression levels of EGFR and STARD4 in HepG2-P and HepG2-LR cells were measured by quantitative reverse transcription PCR. **(E)** The mRNA expression levels of EGFR and STARD4 in Huh7-P and Huh7-LR cells were measured by quantitative reverse transcription PCR. **(F)** The protein expression levels of STARD4, p-EGFR, p-PI3K, and p-AKT in HepG2-P, HepG2-LR, Huh7-P, and Huh7-LR cells were analyzed by western blotting assay. **(G)** Immunofluorescence assays detected the protein abundance of p-EGFR and STARD4 in HepG2-P and HepG2-LR cells. The data were shown as mean ± standard deviation (*n* = 3). ∗*P* < 0.05, ∗∗*P* < 0.01, and ∗∗∗*P* < 0.001 versus the corresponding control.

Drug resistance has always been a huge challenge for target therapy of HCC.^33^ To investigate the molecular mechanism of lenvatinib resistance, we successfully developed two LR HCC cell lines named HepG2-LR and Huh7-LR. Compared with parent cells, both HepG2-LR cells and Huh7-LR cells became less sensitive to lenvatinib with a 6.33-times and 6.26-times increase of IC50 respectively (Fig. 5A). Our study also confirmed that LR HCC cells exhibited higher proliferation (Fig. 5B), migration, and invasion (Fig. 5C) abilities under the conditions of lenvatinib (50 μM for HepG2-LR and 10 μM for Huh7-LR), which implicated that HepG2-LR or Huh7-LR cells acquired high resistance to lenvatinib.

To investigate whether STARD4 and EGFR are involved in lenvatinib resistance, we simultaneously detected the mRNA levels of EGFR and STARD4 in LR HCC cells and parent cells. Both EGFR and STARD4 expression were found to be significantly increased in the LR cells (Fig. 5D, E). Higher protein levels of p-EGFR, p-PI3K, p-AKT, and STARD4 were observed in LR HCC cells (Fig. 5F). Consistent with western blotting findings, immunofluorescence experiments confirmed that increased protein abundance of p-EGFR and STARD4 were present in HepG2-LR (Fig. 5G). These observations reveal that STARD4 and EGFRare significantly upregulated and activated in LR HCC cells, suggesting an association of STARD4 and EGFR signaling with lenvatinib resistance.

### STARD4 impairs the anti-tumor effect of lenvatinib

**Figure 6:**
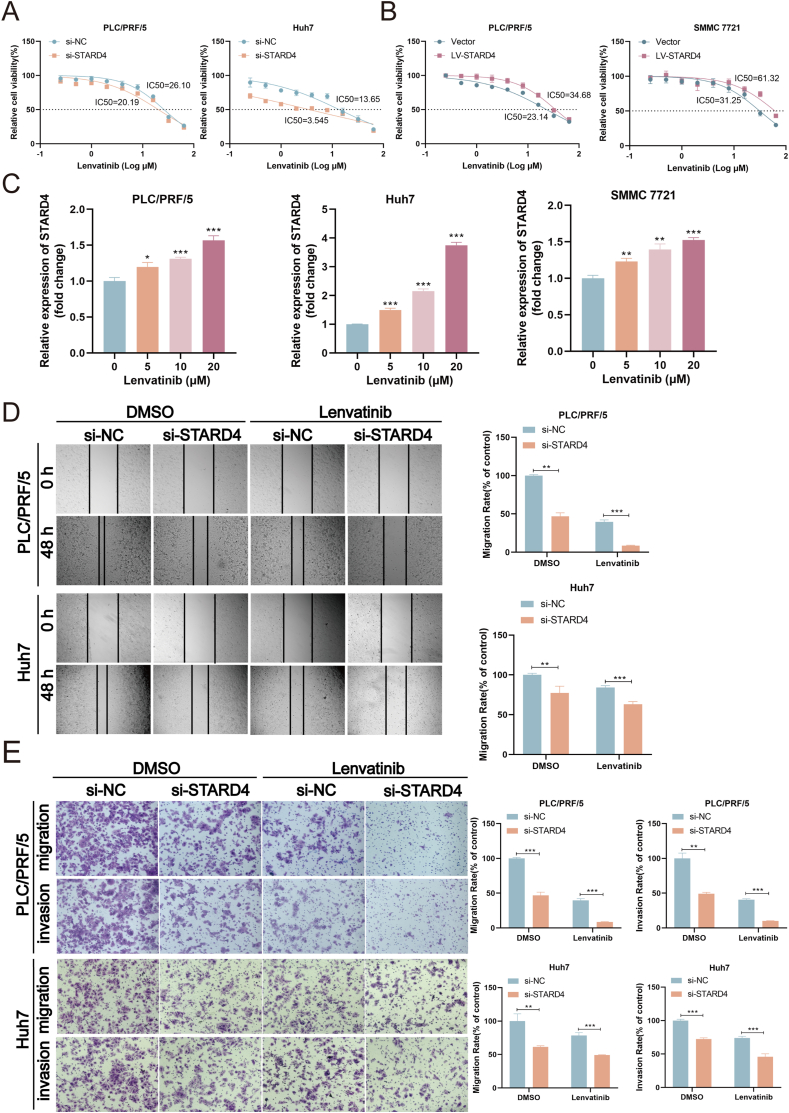
STARD4 impairs the anti-tumor effect of lenvatinib. **(A, B)** The cell viability of STARD4-silencing and STARD4-overexpressing hepatocellular carcinoma cells treated with different concentrations of lenvatinib for 48 h was detected by CCK8 assay. **(C)** The mRNA expression of STARD4 in hepatocellular carcinoma cells treated with lenvatinib was detected by quantitative reverse transcription PCR. **(D, E)** The migration and invasion of hepatocellular carcinoma cells with STARD4 silencing or overexpressing after exposure to lenvatinib (10 μM) were examined by wound healing assay and transwell assay. The data were shown as mean ± standard deviation (*n* = 3). ∗*P* < 0.05, ∗∗*P* < 0.01, and ∗∗∗*P* < 0.001 versus the corresponding control.

To explore the role of STARD4 in lenvatinib resistance, CCK8 assays were used to detect the viability of STARD4 silencing or overexpressing HCC cells under different concentrations of lenvatinib treatment. As shown in Figure 6A, PLC/PRF/5 and Huh7 cells with STARD4 knockdown exhibited higher sensitivity to lenvatinib, according to an obvious decrease of IC_50_. Additionally, overexpression of STARD4 made the HCC cells more resistant to lenvatinib and induced lower growth inhibition in PLC/PRF/5 and SMMC 7721 cells, with a significant increase in IC50 of lenvatinib (Fig. 6B). Next, we identified that the expression of STARD4 in PLC/PRF/5, Huh7, and SMMC 7721 cells showed a gradient increase after treatment with increasing concentration of lenvatinib (Fig. 6C). Furthermore, the knockdown of STARD4 led to a more pronounced inhibition of cell invasion, migration, and colon formation following the exposure to lenvatinib (Fig. 6D, E; Fig. S2C, D). These data together indicate that STARD4 impairs the anti-tumor effect of lenvatinib in HCC cells, while down-regulation of STARD4 enhances sensitivity to lenvatinib.

### The promotion of STARD4 on HCC progression is inhibited by EGFR inhibitor erlotinib

**Figure 7:**
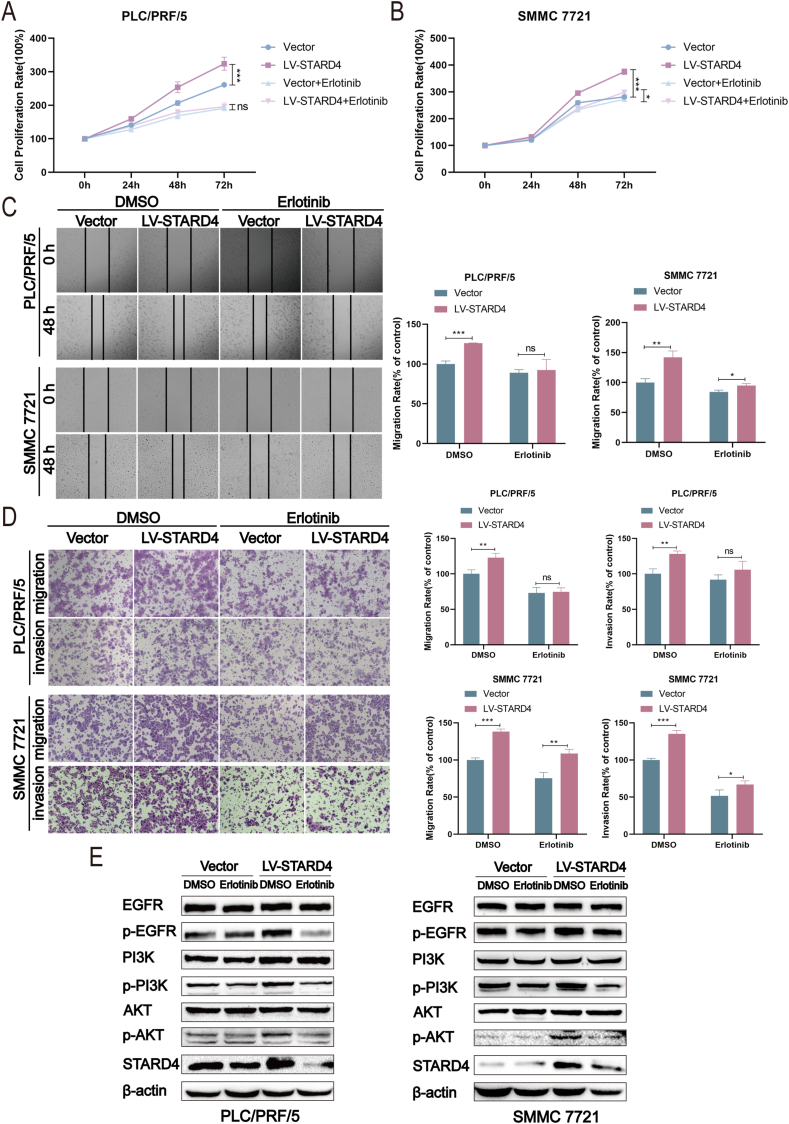
The promotion of STARD4 on the progression of hepatocellular carcinoma is inhibited by the EGFR inhibitor erlotinib. **(A, B)** Erlotinib attenuated the effects of STARD4 overexpressing on the proliferation of hepatocellular carcinoma cells examined by CCK8 assay. **(C, D)** Erlotinib reduced the effects of STARD4 overexpressing on the migration and invasion of hepatocellular carcinoma cells assessed by wound healing assay and transwell assay. **(E)** Effects of erlotinib on the protein levels of p-EGFR, p-PI3K, p-AKT, and STARD4 in hepatocellular carcinoma cells with STARD4 overexpressing and the control cells. The data were shown as mean ± standard deviation (*n* = 3). ∗*P* < 0.05, ∗∗*P* < 0.01, and ∗∗∗*P* < 0.001 versus the corresponding control.

To further elucidate whether STARD4 regulates the progression and lenvatinib resistance of HCC via EGFR activation, we investigated the impact of EGFR inhibitor erlotinib on the tumor-promoting effect of STARD4 in HCC cells. Results from the CCK8 assay, wound healing assay, and transwell assay showed that erlotinib (10 μM) significantly reduced and even in some groups canceled the facilitating effects of STARD4 on cell proliferation (Fig. 7A, B), migration (Fig. 7C, D), and invasion (Fig. 7D) of PLC/PRF/5 and SMMC 7721 cells. In addition, erlotinib significantly enhanced the inhibitory effects of STARD4 knockdown on the migration and invasion of Huh7 and PLC/PRF/5 cells (Fig. S3A, B). Consistent with the above results, overexpression of STARD4 increased protein levels of p-EGFR, p-PI3K, and p-AKT in HCC cells, while erlotinib inhibited this STARD4-induced activation of EGFR/PI3K/AKT signaling pathway. As shown in Figure 7E, erlotinib significantly suppressed the phosphorylation of EGFR, PI3K, and AKT, especially in STARD4 overexpressing HCC cells. Furthermore, erlotinib obviously enhanced the inhibiting effects of STARD4 knockdown on the phosphorylation of EGFR, PI3K, and AKT in PLC/PRF/5 and Huh7 cells (Fig. S3C). Collectively, these results demonstrate that STARD4 exerts tumor-promoting effects through the activation of the EGFR/PI3K/AKT pathway in HCC cells.

### STARD4 knockdown suppresses tumorigenesis and enhances the anti-tumor effect of lenvatinib *in vivo*

**Figure 8:**
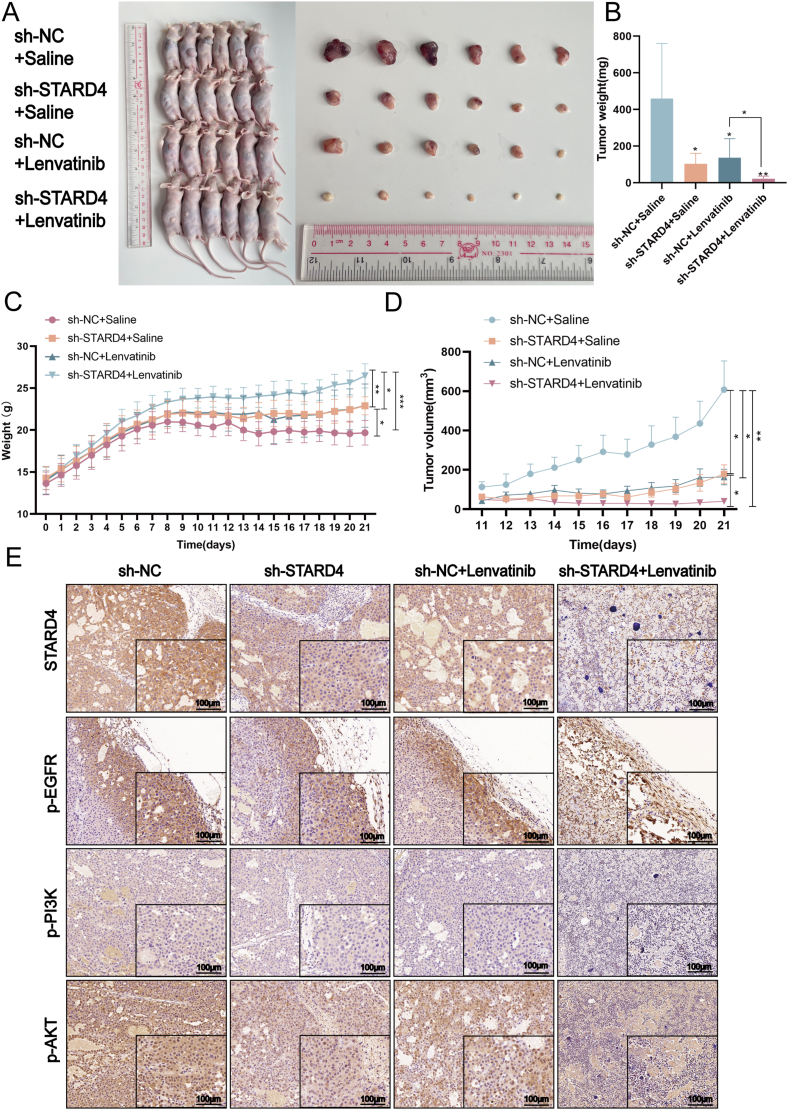
STARD4 knockdown suppresses tumorigenesis and enhances the anti-tumor effect of lenvatinib *in vivo*. **(A)** The images of xenografts in the four groups with different treatments. **(B)** Effect of STARD4 knockdown, lenvatinib, and combination of STARD4 knockdown with lenvatinib on the tumor volume of the nude mice. **(C)** The body weight of the nude mice in each group was weighed daily and shown. **(D)** Effect of STARD4 knockdown, lenvatinib, and combination of STARD4 knockdown with lenvatinib on the tumor weight of the nude mice. **(E)** Immunohistochemical analysis indicated the protein abundance of STARD4, p-EGFR, p-PI3K, and p-AKT in the tumor tissues obtained from the nude mice in different groups. The data were shown as mean ± standard deviation (*n* = 6). ∗*P* < 0.05, ∗∗*P* < 0.01, and ∗∗∗*P* < 0.001 versus the corresponding control.

To further investigate the effect of STARD4 knockdown on tumor growth and sensitivity to lenvatinib, the stably STARD4 silencing PLC/PRF/5 cells and control cells were injected into the right flank of nude mice, and the mice were treated daily with physiological saline or lenvatinib (10 mg/kg) when the tumor reached a volume of approximately 20 mm^3^. The remarkably reduced STARD4 mRNA and protein levels in the STARD4 knockdown stable cell line (sh-STARD4) had been verified by quantitative reverse transcription PCR and Western blot assays (Fig. S3D, E). STARD4 knockdown exhibited a significant suppressing effect on the growth of the subcutaneous tumor and dramatically enhanced the sensitivity of HCC to lenvatinib, according to the tumor weight and volume in each group (Fig. 8A, B, D). The xenograft tumors in the group with a combination of STARD4 knockdown and lenvatinib obtained the lowest growth rate compared with the other groups. In addition, this group of mice was in better condition during the experiment, according to the stable body weight gain (Fig. 8C). Immunohistochemical analysis showed that STARD4 knockdown or lenvatinib either decreased the protein expression of p-EGFR, p-PI3K, and p-AKT in the tumor tissues, while the combination of STARD4 knockdown with lenvatinib exerted a significant synergistic inhibitory effect on the phosphorylation of EGFR, PI3K, and AKT (Fig. 8E). In conclusion, these data from animal studies suggests that STARD4 knockdown suppresses tumorigenesis and enhances the anti-tumor effect of lenvatinib *in vivo* by inhibiting EGFR/PI3K/AKT signaling pathway.

## Discussion

Most tumors demonstrate aberrant activation of lipid metabolism, which promotes tumor progression.^34^ Being fast-proliferating, cancer cells necessitate elevated cholesterol levels for membrane biogenesis and other functional demands.^35^ Nonetheless, excessive cholesterol has been recognized as cytotoxic.^36^^,^^37^ Hence, apart from enhanced synthesis, cancer cells also reprogram cholesterol trafficking and biotransformation to maintain cellular homeostasis.^6^^,^^35^^,^^38^ STARD4, an efficient cholesterol transporter, facilitates cholesterol transportation to the endoplasmic reticulum, fostering the production of cholesteryl ester,^11^ which appears to function as a reservoir of cholesterol that cancer cells can tap under increased demand. Herein, we present the initial comprehensive investigation into the tumor-promoting function and mechanism of STARD4 in HCC.

By analyzing clinical samples from patients diagnosed with HCC, we observed an up-regulation of both mRNA and protein expression of STARD4 in tumor tissues compared with adjacent normal tissues. Additionally, an investigation into the correlation between STARD4 and clinicopathological characteristics revealed a strong association between STARD4 expression and the progression of malignancy in HCC. These findings align with data from the Cancer Genome Atlas, which supports a significant relationship between the expression levels of STARD4 and overall survival time in HCC patients. Consequently, our study indicates that STARD4 holds potential as a biomarker for predicting outcomes in HCC patients. To further elucidate the role of STARD4 in the malignant progression of HCC, we conducted a series of experiments including CCK8, wound healing, transwell, colony formation, tube formation assays, and animal studies. The results consistently demonstrated that down-regulating STARD4 expression effectively attenuated the proliferation, migration, invasion, and angiogenesis capabilities of HCC cells. Conversely, up-regulating the expression of STARD4 enhanced the malignancy of these cells. Notably, these *in vitro* analyses were corroborated by an animal study, which illustrated that STARD4 significantly facilitated the growth of xenograft tumors in nude mice. Collectively, our findings unequivocally establish the tumorigenic role of STARD4 both *in vitro* and *in vivo*.

Given the well-established role of STARD4 as a vital cholesterol transporter, we aimed to explore its impact on free cholesterol levels within HCC cells. Our findings demonstrated that depletion of STARD4 led to an augmentation in cholesterol accumulation within the plasma membrane, while overexpression of STARD4 exerted the opposite effect. Consistent with our observations, previous research has also documented that down-regulation of STARD4 heightened intracellular free cholesterol levels and impeded sterol transport in human U2OS osteosarcoma cells.^14^ Moreover, it has been reported that STARD4 enhances the activity of ACAT-1, thereby facilitating cholesterol esterification.^11^ Building upon these studies, we propose a hypothesis suggesting that STARD4 may shuttle cholesterol to the endoplasmic reticulum, consequently triggering cholesteryl ester production and reducing the distribution of free cholesterol within the plasma membrane of HCC. Notably, ACAT-1 has been deemed a pro-tumorigenic factor in HCC and pancreatic cancer^38^; however, whether ACAT-1 is involved in the tumor-promoting effect of SDTARD4 necessitates further investigation.

Recent studies have demonstrated that the depletion of cholesterol from the plasma membrane can trigger EGFR dimerization and phosphorylation, while also delaying endocytosis.^28^ This phenomenon leads to the activation of downstream MAPK and PI3K pathways. Pike and Casey^39^ revealed that the depletion of cholesterol affected the functionality of the EGF receptor due to the release of the receptor from inhibitory constraints imposed by its localization in lipid rafts. These findings motivated us to investigate whether STARD4 could promote EGFR signaling in HCC cells. In our study, we discovered a substantial positive correlation between STARD4 and EGFR mRNA expression in tumor tissue samples obtained from HCC patients. Moreover, the knockdown of STARD4 resulted in decreased EGFR phosphorylation in HCC cells, while its overexpression led to increased phosphorylation. These observations highlight the significant role of STARD4 in the activation of EGFR. Subsequently, we employed MβCD to eliminate cholesterol from the cell membrane and found that EGFR phosphorylation increased in both control and STARD4 knockdown cells. However, the impact of STARD4 knockdown on EGFR phosphorylation became insignificant after MβCD treatment. These results suggest that STARD4 enhances EGFR phosphorylation by affecting cholesterol levels in the plasma membrane. To further explore the key downstream pathway of EGFR, we observed that the silencing of STARD4 inhibited the phosphorylation of PI3K and AKT. In conclusion, our findings demonstrate that STARD4 promotes the activation of the EGFR/PI3K/AKT signaling pathway in HCC cells by regulating cholesterol homeostasis.

Although the role of EGFR signaling in lenvatinib resistance has been supported by increasing evidence,^25^^,^^32^ few studies have investigated the underlying mechanism of EGFR activation responsible for drug resistance. Our study has demonstrated a significant up-regulation of STARD4 expression and activation of the EGFR/PI3K/AKT signaling pathway in LR HCC cells. Additionally, we have identified that STARD4 impairs the anti-tumor effect of lenvatinib in HCC, while the down-regulation of STARD4 enhances sensitivity to lenvatinib. Animal studies also confirmed that a combination of STARD4 and lenvatinib had a significant synergistic inhibitory effect on the growth of the xenograft tumors and activation of EGFR signal. EGFR inhibitor erlotinib could suppress the promotion of STARD4 on the progression of HCC, which further reinforced the idea that STARD4 exerts tumor-promoting effects and enhances the resistance to lenvatinib through activation of EGFR/PI3K/AKT pathway in HCC cells. Moreover, Western blot analysis has shown that erlotinib can decrease the protein level of STARD4, suggesting that the EGFR signal might regulate the expression of STARD4. We hypothesize the existence of a positive feedback regulation between STARD4 and EGFR, and future investigations will be conducted to confirm this hypothesis.

In conclusion, the current study provides evidence that STARD4 promotes the proliferation, metastasis, and lenvatinib resistance of HCC by modulating cholesterol homeostasis and subsequently activating the EGFR/PI3K/AKT pathway. These findings propose that STARD4 holds potential as a valuable molecular biomarker for predicting lenvatinib resistance and as a promising therapeutic target for HCC treatment.

## CRediT authorship contribution statement

**Mengting Liu:** Conceptualization, Data curation, Formal analysis, Investigation, Methodology, Validation, Writing – original draft. **Yixin Liu:** Conceptualization, Data curation, Formal analysis, Investigation, Software, Validation, Writing – original draft, Writing – review & editing. **Jiahui Zheng:** Data curation, Validation, Writing – review & editing. **Xiangping An:** Formal analysis, Investigation. **Jiayong Wen:** Conceptualization, Data curation, Validation. **Fengchi Zhu:** Conceptualization, Software, Visualization. **Jin Jia:** Conceptualization, Formal analysis, Validation. **Dan Guo:** Funding acquisition, Methodology, Project administration, Writing – review & editing. **Nana Chen:** Conceptualization, Formal analysis, Funding acquisition, Methodology, Project administration, Supervision.

## Ethics declaration

The animal experiments and treatments were approved by the Institutional Animal Care and Use Committee (IACUC) of Southern Medical University (SMU), Guangzhou, China (approval No. L2018142). Informed consent was obtained where applicable. The clinical tissues were obtained with informed consent and the relative research was approved by the Medical Ethics Committee of Nanfang Hospital of Southern Medical University (approval No. NFEC-2023-258).

## Funding

This work was supported by the 10.13039/501100021171Guangdong Basic and Applied Basic Research Foundation of China (No. 2023A1515012595, 2024A1515012743), 10.13039/501100010112Nanfang Hospital Dean's Fund (No. 2022A010), 10.13039/100007452Wu Jieping Medical Foundation (No. 320.6750.2023-06-18), and Hospital Pharmacy Research Foundation of the Guangdong Liver Disease Association of China (No. 2023gdsgzbzd01).

## Conflict of interests

The authors declared no conflict of interests.
