# Supplementary material for: Targeting STARD4/EGFR axis inhibits growth and overcomes lenvatinib resistance in hepatocellular carcinoma
Source: Genes Dis. 2025 Feb 15;12(6):101556. doi: 10.1016/j.gendis.2025.101556 (PMC12359158; doi:10.1016/j.gendis.2025.101556)
Supplement: Multimedia component 1 [file mmc1.docx]

**Table S1 Correlation between STARD4 and clinicopathological characteristics**

**in 52 HCC patients**

| Clinical features | STARD4 Expression | | P Value |
| --- | --- | --- | --- |
|  | High | Low |  |
| Total number | 26 | 26 |  |
| Age (years) |  |  |  |
| ＜50 | 12 | 8 | 0.254 |
| ≥50 | 14 | 18 |  |
| Gender |  |  |  |
| Male | 25 | 25 | 1.00 |
| Female | 1 | 1 |  |
| Tumor size (cm) |  |  |  |
| ＜5 | 15 | 22 | 0.032* |
| ≥5 | 11 | 4 |  |
| AFP (ng/ml) |  |  |  |
| ＜200 | 15 | 22 | 0.032* |
| ≥200 | 11 | 4 |  |
| Hepatitis |  |  |  |
| without | 1 | 9 | 0.005* |
| with | 25 | 17 |  |
| TNM stage |  |  |  |
| I+II | 10 | 25 | ＜0.001* |
| III+IV | 16 | 1 |  |
| Microvascular infiltration |  |  |  |
| without | 4 | 20 | ＜0.001* |
| with | 22 | 6 |  |

**Figure S1 Detection of SATRD4 knockdown or overexpression efficiency and the effect of STARD4 knockdown on angiogenesis**


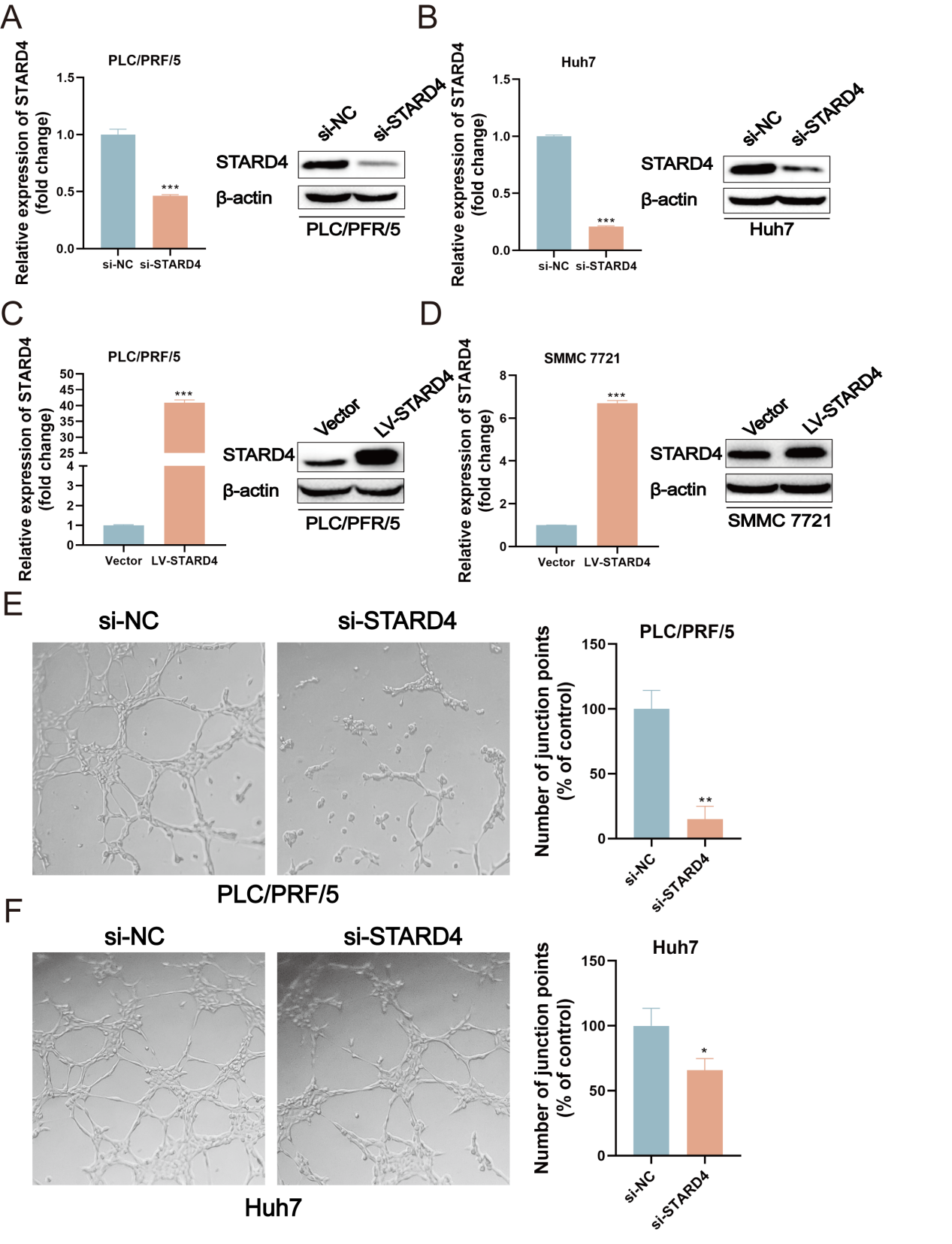


**Figure S1.** **Detection of SATRD4 knockdown or overexpression efficiency and the effect of STARD4 knockdown on angiogenesis.** (A-B) qRT-PCR and western blot indicated the expression of STARD4 in Huh7 and PLC/PRF/5 transfected with siRNA against STARD4. (C-D) qRT-PCR and western blot indicated the expression of STARD4 in SMMC 7721 and PLC/PRF/5 transfected with overexpression plasmids with STARD4.(E-F) Angiogenesis formation assay examined the pro-angiogenic capacity of HCC cells after STARD4 knockdown. The data were shown as means±SD (n=3). *, p<0.05; **, p<0.01; ***, p<0.001; compared with the corresponding control.

**Figure S2 Detection of stable transfection overexpression efficiency of SATRD4 and effect of STARD4 knockdown on lenvatinib sensitivity**


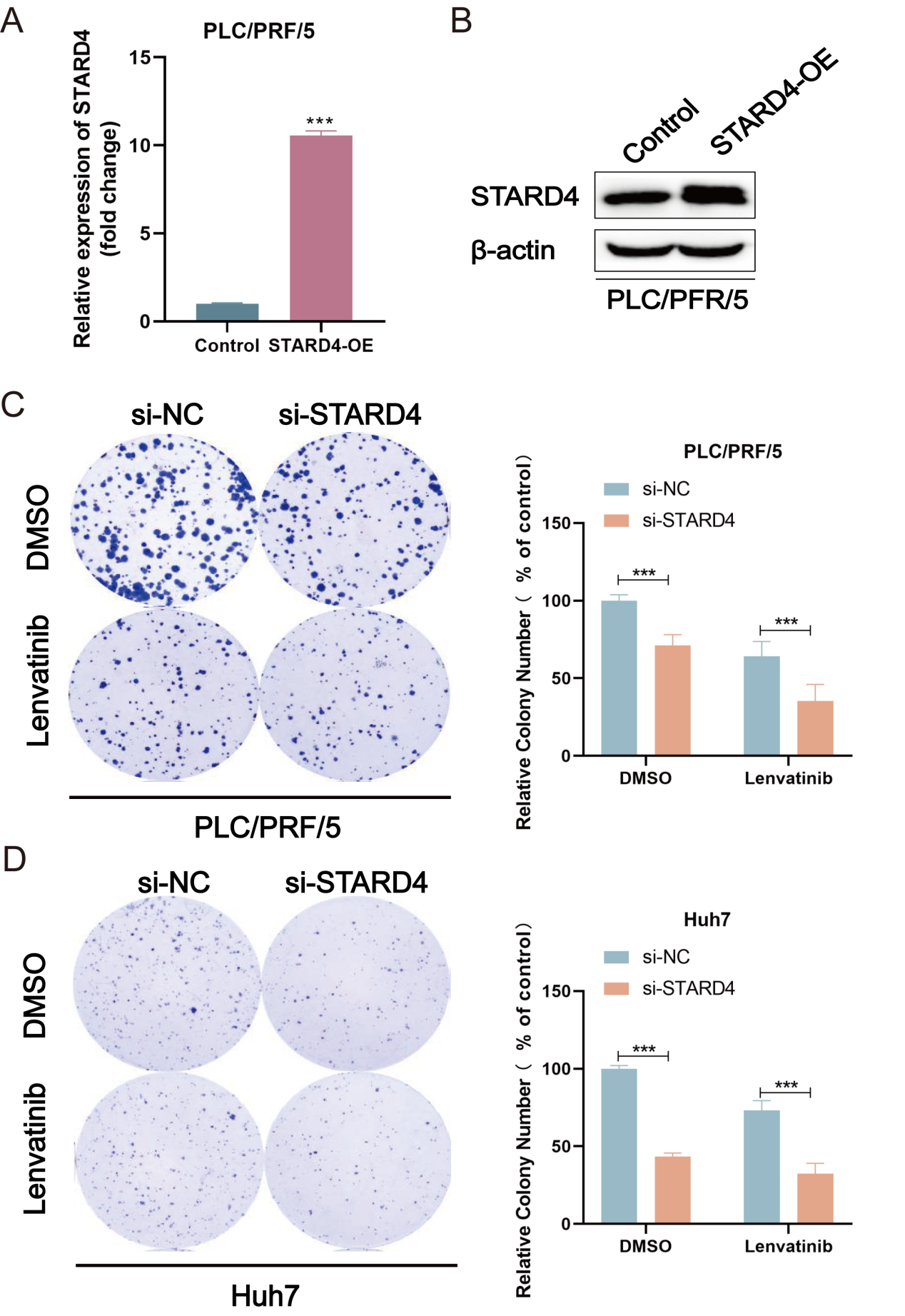


**Figure S2.** **Detection of stable transfection overexpression efficiency of SATRD4 and effect of STARD4 knockdown on lenvatinib sensitivity.** (A-B) qRT-PCR and western blot indicated the expression of STARD4 in stable PLC/PRF/5 transfected with overexpression plasmids with STARD4. (C-D) The multiplication of HCC cells added with lenvatinib(10μM) after STARD4 silencing estimated by colony formation assay. The data were shown as means±SD (n=3). *, p<0.05; **, p<0.01; ***, p<0.001; compared with the corresponding control.

**Figure S3 Effects of EGFR inhibitors on STARD4 knockdown on HCC cells and detection of efficiency of STARD4 stable knockdown cells**


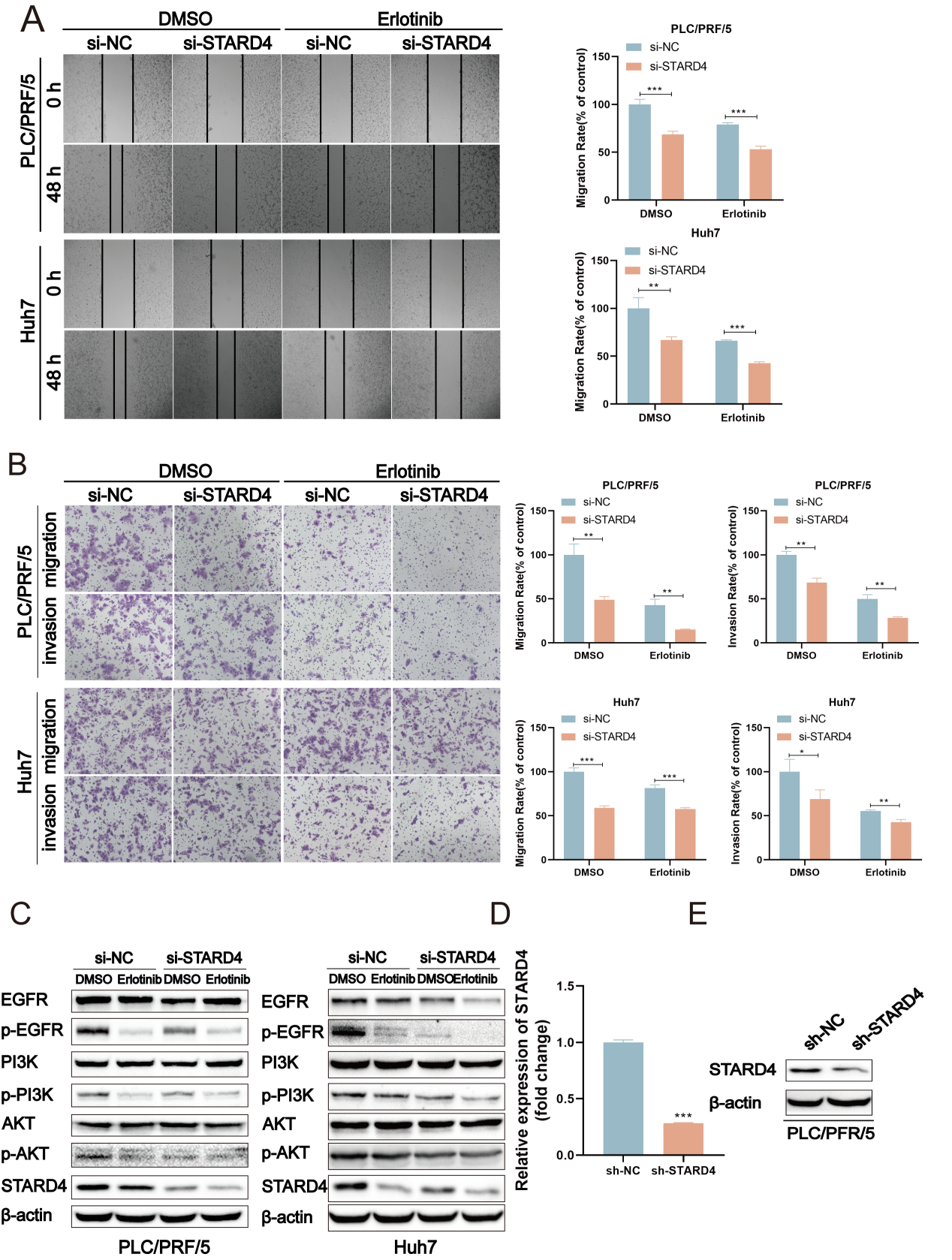


**Figure S3.** **Effects of EGFR inhibitors on STARD4 knockdown on HCC cells and detection of efficiency of STARD4 stable knockdown cells.** (A-B)The migration and invasion of HCC cells added with erlotinib(10μM) after STARD4 silencing estimated by Wound healing and Transwell assay. (C)The protein expression levels of STARD4, p-EGFR, p-PI3K, p-AKT in HCC cells added with erlotinib(10μM) after STARD4 silencing. (D-E) qRT-PCR and western blot indicated the expression of STARD4 in stable PLC/PRF/5 transfected with shRNA against STARD4. The data were shown as means±SD (n=3). *, p<0.05; **, p<0.01; ***, p<0.001; compared with the corresponding control.
